# Using Permutations for Hierarchical Clustering of Time Series

**DOI:** 10.3390/e21030306

**Published:** 2019-03-21

**Authors:** Jose S. Cánovas, Antonio Guillamón, María Carmen Ruiz-Abellón

**Affiliations:** Departamento de Matemática Aplicada y Estadística, Universidad Politécnica de Cartagena, 30202 Cartagena, Spain

**Keywords:** time series clustering, permutation entropy, time series dependency, hierarchical clustering, mutual information

## Abstract

Two distances based on permutations are considered to measure the similarity of two time series according to their strength of dependency. The distance measures are used together with different linkages to get hierarchical clustering methods of time series by dependency. We apply these distances to both simulated theoretical and real data series. For simulated time series the distances show good clustering results, both in the case of linear and non-linear dependencies. The effect of the embedding dimension and the linkage method are also analyzed. Finally, several real data series are properly clustered using the proposed method.

## 1. Introduction and Main Definitions

The goal of time series clustering is to split a set of time series into homogeneous groups, that is, similar time series should lie in the same cluster. However, there are many distances to measure the degree of similarity between two time series, depending on the clustering objectives. The most popular are the Euclidean and correlation-based distances. However, if we want to cluster time series in shape, it has been proved that Dynamic Time Warping distance (see [1]) is more appropriate. Some other distance measures are the short time series given in [2], the Kullback-Leibler studied in [3] or the recent copula-based distance introduced in [4].

Interesting surveys in the field can be found in [5,6], where three different types of clustering approaches are distinguished: shape-based, feature-based and model-based approach. In most cases, the procedures only take into account univariate features of the time series and do not consider the possible relationships among them. Therefore, these methods are useful in the case of independent time series and when the objective is to group them looking at the similarity of their univariate models. It is well known that the selection of a suitable distance measure mainly depends on the objective of clustering. Typically three objectives can be distinguished [5]: finding similar time series in time, finding similar time series in shape and finding similar time series in change (structural similarity). In the first case, the Euclidean distance on raw time series or the Wavelet Transform distance are proper for the first goal. The distances based on Pearson’s correlation are usually considered of this type, even though the aim is to put the time series that are correlated in the same cluster. In the second case, where the time of occurrence of patterns in not important, the use of elastic methods like the Dynamic Time Warping distance is highly encouraged. In the third case, where the aim is to cluster time series with similar structure, autocorrelation-based measures are appropriate. All these similarity (dissimilarity) measures have been applied to some simulated data and compared to our proposed measures to illustrate that they are not proper for clustering time series by dependency. Many of these clustering methods are available in the R package TSclust, developed by Montero and Vilar [7].

Clustering time series by dependency has recently been analyzed in [8,9,10]. In these works, the goal is to cluster the time series according to their degree of dependency. The two former assume that the vector of time series is generated by a Dynamic Factor Model where some factors affect different groups of series. The latter proposes the generalized cross correlation as a general measure of linear association between two time series.

There are a wide range of papers dealing with the applications of ordinal patterns in time series analysis. For example, the utility of permutations to detect structural changes in time series has been widely studied and applied to real data such as speech signals [11], electroencephalogram signals [12,13,14,15] or economic and seismic data [16]. On the other hand, the discriminative power of ordinal pattern statistics and symbolic dynamics to classify cardiac biosignals has been evaluated in [17], whereas [18] uses ordinal patterns to detect and locate change points in the time series and classify the segments with similar dynamics. In this context, Ref. [19] proposes a new metric called Ordinal Synchronization to evaluate the level of synchronization between time series by means of a projection into ordinal patterns.

The aim of the present paper is to illustrate the utility of symbolic dynamic (the time series are codified by means of permutations) in clustering time series by dependency, where the main contribution respect to the works mentioned above are the absence of assumptions and the detection of linear and non-linear dependencies. For that, we have based on the work developed by Ruiz-Abellon et al. [20], where the next three aspects are combined: the time series codification by means of permutations, the distance measures among time series and different linkages for hierarchical clustering.

In this paper we will consider the labeling by permutations technique jointly with some measures which allow us to group different time series, or at least to state what time series are closer in the sense of the considered measure. Let us introduce the basic notation.

Several authors have pointed out that permutations can be a good tool to study time series. In [21] (see also [22,23,24]) the notion of *permutation entropy* has been used for analyzing time series. Let us recall a few notions on permutation labeling of a time series.

Let ${\left(x_{n}\right)}_{n=1}^{T}$, T∈N∪{∞}, be a sequence which comes from real or simulated data. For m≥2, let $S_{m}$ be the group of permutations of length *m*, whose cardinality is m!. Let $x_{m}\left(r\right)=\left(x_{r},x_{r+1},\ldots ,x_{r+m-1}\right)$, 1≤r<T−m+1, be a sliding window taken from the sequence ${\left(x_{n}\right)}_{n=1}^{T}$. We say that the window $x_{m}\left(r\right)$ is of π–type, $\pi \in S_{m}$, if and only if $\pi =(i_{1},i_{2},\ldots ,i_{m})$ is the unique element of $S_{m}$ satisfying the two following conditions:

(c1) $x_{r+i_{1}}\leq x_{r+i_{2}}\leq \ldots x_{r+i_{m}}.$
(c2) $i_{s-1}<i_{s}$ if $x_{r+i_{s-1}}=x_{r+i_{s}}$, with 2≤s≤m.

The positive integer *m* is usually known as *embedding dimension*. Fix r<T−m+1. For each $\pi \in S_{m}$, the probability of occurrence of π is given by:

$$p\left(\pi \right)=\frac{\#\{x_{m}\left(j\right),j=1,2,\ldots ,r:x_{m}\left(j\right)\;\mathrm{is}\;\mathrm{of}\;\pi –\mathrm{type}\}}{r}.$$

A permutation $\pi \in S_{m}$ with p(π)>0 for some r<T−m+1 is called an *admissible permutation* of ${\left(x_{n}\right)}_{n=1}^{T}$. It is clear that permutations are linked to data series complexity. For instance, a periodic or increasing time series has at most a finite number of admissible permutations which are bounded for any embedding dimension *m*. Conversely, it is also clear that a big enough i.i.d noise should admit any permutation of length *m*. On the other hand, for piecewise monotone maps, it is proved that topological entropy, a useful tool to decide whether a deterministic time series is complicated, can be computed by using permutations [25,26].

The above-described codification can be extended in a direct way when we have two dimensional time series. Let ${\left(x_{n}\right)}_{n=1}^{T}$ and ${\left(y_{n}\right)}_{n=1}^{T}$ be two real time series and let ${\left(z_{n}\right)}_{n=1}^{T}$ be the corresponding two-dimensional time series with $z_{n}=\left(x_{n},y_{n}\right)$, for all n=1,…,T. Let $z_{m}\left(r\right)=\left(x_{m}\left(r\right),y_{m}\left(r\right)\right)$, 1≤r<T−m+1, be a two-dimensional sliding window taken from the sequence ${\left(z_{n}\right)}_{n=1}^{T}$. The window $z_{m}\left(r\right)$ is said to be of type $\pi_{i}\times \pi_{j}$ if and only if $x_{m}\left(r\right)$ is a $\pi_{i}$–type and $y_{m}\left(r\right)$ is of $\pi_{j}$ –type. After the codifying process, all of the empirical information is collected in a contingency table, where $O_{i,j}$ denotes the observed frequency of the symbol $\pi_{i}\times \pi_{j}$ and as usual, the relative frequency is given by:

$$p\left(\pi_{i}\times \pi_{j}\right)=\frac{O_{i,j}}{T-m+1},$$

for 1≤i,j≤m!. The contingency table is shown below.


$\left(x_{n}\right)/\left(y_{n}\right)$
$\pi_{1}$–type$\pi_{2}$–type…$\pi_{m!}$ –type
$\pi_{1}$–type
$O_{1,1}$

$O_{1,2}$
…
$O_{1,m!}$

$O_{1•}$
$\pi_{2}$–type
$O_{2,1}$

$O_{2,2}$
…
$O_{2,m!}$

$O_{2•}$
………………$\pi_{m!}$–type
$O_{m!,1}$

$O_{m!,2}$
…
$O_{m!,m!}$

$O_{m!•}$


$O_{•1}$

$O_{•2}$
…
$O_{•m!}$



The above contingency table was used in [27] to decide whether two time series were independent or not. Among others, the Pearson’s chi–square statistic was used for the above contingency table given by:

$$\chi^{2}=\sum_{i=1}^{m!}\sum_{j=1}^{m!}\frac{{\left(O_{i,j}-e_{i,j}\right)}^{2}}{e_{i,j}},$$

$e_{i,j}$ denotes the expected frequencies under the hypothesis of independence and is given by:

$$e_{i,j}=\frac{O_{i•}\;O_{•j}}{T-m+1}.$$

Hence, we can define the Crammer’s V measure as follows, see [28]:

$$V\left(x_{n},y_{n}\right)=\sqrt{\frac{\chi^{2}}{\left(T-m-1)(m!-1\right)}}.$$

Values of Cramer’s V close to zero means no association (independency) and close to one mean strong association (dependency). Cramer’s V measure allows us to define the “distance”:

$$D_{V}\left(x_{n},y_{n}\right)=1-V\left(x_{n},y_{n}\right).$$

It is unclear whether $D_{V}$ is a metric, but as we will show later, it is a good help in clustering data series. Before that, we will introduce another measure based on mutual information measures.

Given two discrete random variables *X* and *Y*, the mutual information coefficient (see [29]) is defined by:

$$I\left(X,Y\right)=\sum_{i=1}^{n}\sum_{j=1}^{m}p\left(x_{i},y_{j}\right)\log\frac{p(x_{i},y_{j})}{p_{1}\left(x_{i}\right)p_{2}\left(y_{j}\right)}$$

where $p(x_{i},y_{j})$ is the join probability function of (X,Y) and $p_{1}\left(x_{i}\right)$ and $p_{2}\left(y_{j}\right)$ are the marginal probability functions of *X* and *Y*, respectively. The mutual information coefficient can be computed using the concept of Shannon entropy (see [30]) by:

I(X,Y)=H(X)+H(Y)−H(X,Y)

where

$$H\left(X\right)=-\sum_{i=1}^{n}p_{1}\left(x_{i}\right)\log p_{1}\left(x_{i}\right)$$

and

$$H\left(Y\right)=-\sum_{j=1}^{m}p_{2}\left(y_{j}\right)\log p_{2}\left(y_{j}\right)$$

are the Shannon entropies of *X* and *Y*, respectively, and

$$H\left(X,Y\right)=-\sum_{i=1}^{n}\sum_{j=1}^{m}p\left(x_{i},y_{j}\right)\log p\left(x_{i},y_{j}\right)$$

is the Shannon entropy of (X,Y). The mutual information coefficient is a dependency measure because I(X,Y)=0 if and only if *X* and *Y* are independent. Moreover, it is symmetric and non-negative, but there is not a fixed upper bound. Hence, we can consider the metric given in [29]

$$D\left(X,Y\right)=1-\frac{I(X,Y)}{\max\{H(X),H(Y)\}},$$

which is a metric because it is non–negative, symmetric and holds the triangular property. Applied to the codified time series it reads as:

$$D\left(x_{n},y_{n}\right)=1-\frac{\sum_{\pi_{i}\in S_{m}}\sum_{\pi_{j}\in S_{m}}\frac{O_{i,j}}{T-m+1}\log\frac{\left(T-m+1\right)O_{i,j}}{O_{i•}O_{j•}}}{\max\left\{-\sum_{\pi_{i}\in S_{m}}\frac{O_{i•}}{T-m+1}\log\frac{O_{i•}}{T-m+1},-\sum_{\pi_{j}\in S_{m}}\frac{O_{j•}}{T-m+1}\log\frac{O_{j•}}{T-m+1}\right\}}$$

Remark that the efficiency of the Pearson’s chi-square statistic (applied to symbolic dynamic) to detect linear and non-linear dependencies between two time series was illustrated in [27]. Therefore, a dissimilarity measure based on this statistic can be useful to develop a new clustering approach. Analogously, the efficiency of the mutual information coefficient for detecting dependencies was shown, among others, in [29], so a dissimilarity measure combining symbolic dynamic and the mutual information coefficient can be proposed as a good tool to cluster time series by dependency.

In the next section, different scenarios are simulated: linear dependency among time series using several models (the logistic map, uniformly distributed noise and autoregressive process); non-linear dependency for deterministic systems and non-linear dependency for non-deterministic systems. Additionally, simulations were carried out for different embedding dimensions and three hierarchical linkages (single, complete and average) to analyze their effect on the resulting dendrograms.

## 2. Synthetic Experiments

### 2.1. Linear Dependence

We consider data series by following the system of difference equations as follows:

(1) $$x_{n+1}^{i}=\sum_{j=1}^{k}\lambda_{ij}f_{j}\left(x_{n}^{j}\right),$$

where n≥0, k≥3, $f_{j}$ are real functions for 1≤j≤k, and for 1≤i,j≤k, we have that $\lambda_{ij}\geq 0$ and

$$\sum_{j=1}^{k}\lambda_{ij}\leq 1.$$

These maps have been introduced as a model for migration in population dynamics (see e.g., [31] and some references therein). In principle, if $\lambda_{ij}=\lambda_{ji}=0$, then there is no relationship between sequences $x_{n}^{i}$ and $x_{n}^{j}$. Of course, we are assuming that some of them are not completely independent. Note that if both sequences are independent and are generated by the same deterministic map *f* with the same initial condition, then they are the same sequence indeed.

The experiments we have done are as follows. We generate several data series and apply $D_{V}$ and *D* to cluster them. The smaller values of $D_{V}$ or *D* we get, the closer the two data series are in the sense of these measures.

Below, we summarize the experiments we have done.

We take k=5 and consider the matrix:

$$\Lambda_{1}=\left(\lambda_{ij}\right)=\left(\begin{array}{ccccc} 0.1 & 0.9 & 0 & 0 & 0\\ 0.9 & 0.1 & 0 & 0 & 0\\ 0 & 0 & 0.2 & 0.8 & 0\\ 0 & 0 & 0.8 & 0.2 & 0\\ 0 & 0 & 0 & 0 & 1 \end{array}\right).$$

**Figure 1:**
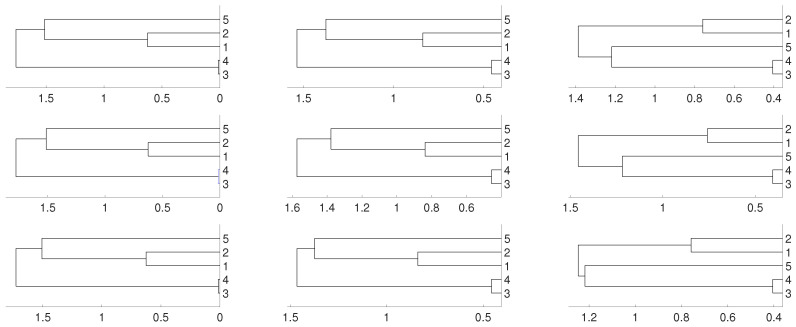
Dendrograms for distance $D_{V}$ of the logistic time series for embedding dimension m=2 (**left** column), m=3 (**central** column) and m=4 (**right** column), and three linkages, average (**first** row), complete (**central** row) and single (**bottom** row). To improve the visibility we write 1 for $x_{n}^{1}$ and so on. We see a closed connection between $x_{n}^{3}$ and $x_{n}^{4}$ as expected, then between $x_{n}^{1}$ and $x_{n}^{2}$. The difference appears with $x_{n}^{5}$ which is more close to $x_{n}^{1}$ and $x_{n}^{2}$ for m=2,3 and to $x_{n}^{3}$ and $x_{n}^{4}$ for m=4.

**Figure 2:**
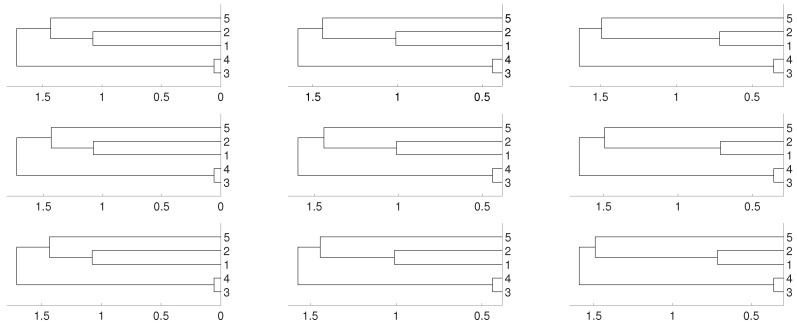
Dendrograms for distance *D* of the logistic time series for embedding dimension m=2 (**left** column), m=3 (**central** column) and m=4 (**right** column), and three linkages, average (**first** row), complete (**central** row) and single (**bottom** row). To improve the visibility we write 1 for $x_{n}^{1}$ and so on. We see a closed connection between $x_{n}^{3}$ and $x_{n}^{4}$ as expected, then between $x_{n}^{1}$ and $x_{n}^{2}$. In all cases $x_{n}^{5}$ is closer to $x_{n}^{1}$ and $x_{n}^{2}$.

According to this matrix, sequences $x_{n}^{1}$ and $x_{n}^{2}$ are linked, as well as $x_{n}^{3}$ and $x_{n}^{4}$, while sequence $x_{n}^{5}$ is isolated. We consider several possibilities for $f_{j}$, 1≤j≤5. Firstly, we take $f_{j}\left(x\right)=4x\left(1-x\right)$ (the logistic map) and random initial conditions. The data length is 10,000 and we consider different embeddings m=2,3,4. We consider distances $D_{V}$ and *D* and three different linkages for the hierarchical method (single, complete and average). This is the general procedure along the paper. As we can see, there are not significative differences in applying the three different hierarchical linkages. In this example, and as it is expected, $x_{n}^{3}$ and $x_{n}^{4}$ are linked together as well as $x_{n}^{1}$ and $x_{n}^{2}$, but the result shows a deep link between variables $x_{n}^{3}$ and $x_{n}^{4}$. Figure 1 and Figure 2 show the results we obtain in detail.

**Figure 3:**
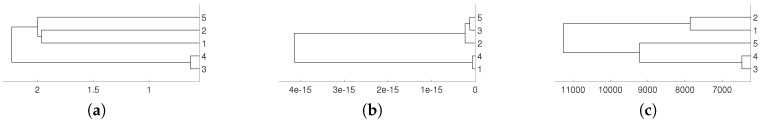
Dendrograms for the logistic time series given by the system defined in (1), average linkage and distances: (**a**) Pearson’s correlation, (**b**) Wavelet Transform and (**c**) Dynamic Time Warping. To improve the visibility we write 1 for $x_{n}^{1}$ and so on.

In order to compare the results of the proposed approach with some traditional ones, Figure 3 shows the dendrograms obtained for system (1) and the logistic time series using the Wavelet Transform, Pearson’s correlation and Dynamic Time Warping distances. For the correlation-based distance the right clustering is achieved as expected, because the dependency among the time series given by system (1) is linear. However, the Wavelet Transform distance is not able to detect the correct relationships (recall that the objective of this distance is to find similar time series in time). Even though the Dynamic Time Warping distance provides the expected clustering results in this case, we will see later an example where it does not.

**Figure 4:**
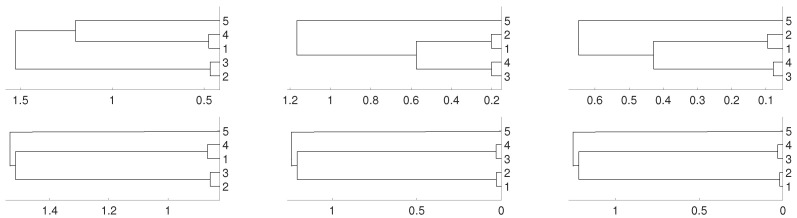
Dendrograms for distance *D* (first row) of a uniform i.i.d. time series for embedding dimension m=2 (**left** column), m=3 (**central** column) and m=4 (**right** column), and average as linkage method. In the second row we show the same for distance $D_{V}$. To improve the visibility we write 1 for $x_{n}^{1}$ and so on.

Then, we repeat the experiments when $f_{j}$ is an i.i.d. uniformly distributed noise for 1≤j≤5. To avoid repetitive graphics we only use the average linkage in Figure 4 for obtaining the dendrograms. No significative differences are obtained when the other two linkages (single and complete) are used instead of the average one. Figure 4 shows the clustering results obtained for both distances $D_{V}$ and *D*. We obtain the same results as in the previous purely deterministic case for m=3 and m=4. However, for m=2 the actual relationships are not properly detected.

**Figure 5:**
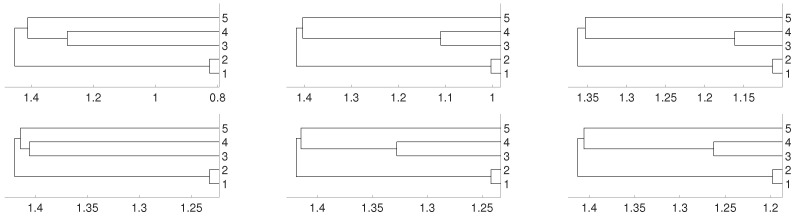
Dendrograms for distance *D* (first row) of an autoregressive time series for embedding dimension m=2 (**left** column), m=3 (**central** column) and m=4 (**right** column), and average linkage method. In the second row we show the same for distance $D_{V}$. To improve the visibility we write 1 for $x_{n}^{1}$ and so on.

Again, we repeat the experiment when $f_{j}\left(x\right)=0.9x+\varepsilon$, where ε is an i.i.d. uniformly distributed noise, that is, $f_{j}$ is an autoregressive process. The dendrograms shown in Figure 5 give us the same result as in the two previous cases.

### 2.2. Non Linear Dependence: Deterministic Systems

**Figure 6:**
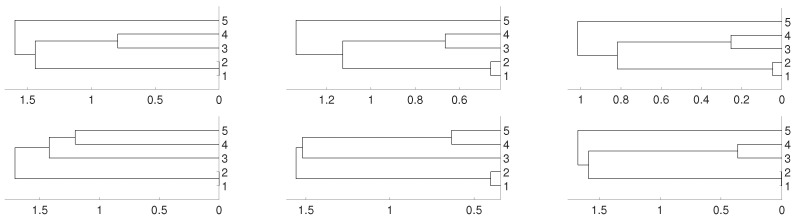
Dendrograms of system (2) for distance *D* (first row) for embedding dimension m=2 (**left** column), m=3 (**central** column) and m=4 (**right** column), and average linkage method. In the second row we show the same for distance $D_{V}$. To improve the visibility we write 1 for $x_{n}^{1}$ and so on.

Now, we consider non linear time series constructed by deterministic systems. We consider the system:

(2) $$\left\{\begin{array}{c} x_{n+1}^{1}=2.25x_{n}^{1}\left(1-x_{n}^{2}\right)\\ x_{n+1}^{2}=x_{n}^{1}\\ x_{n+1}^{3}=x_{n}^{4}\\ x_{n+1}^{4}=x_{n}^{3}\left(1-x_{n}^{4}\right)\\ x_{n+1}^{5}=4x_{n}^{5}\left(1-x_{n}^{5}\right) \end{array}\right.$$

with initial conditions 0.45 for all the variables. We consider a sample of 10,000 points and show the results with the average linkage method. We obtain that $x_{n}^{1}$ and $x_{n}^{2}$ are linked as expected, then $x_{n}^{3}$ and $x_{n}^{4}$, as one can expect given the system shape. Dendrograms are shown in Figure 6. Note that for the *D* distance, the expected results are obtained using different embeddings, whereas for the $D_{V}$ distance, only with m=4.

**Figure 7:**
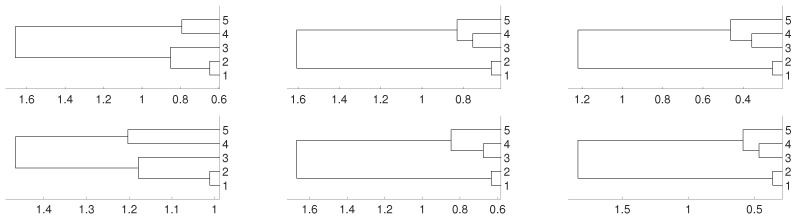
Dendrograms of system (3) for distance *D* (first row) for embedding dimension m=2 (**left** column), m=3 (**central** column) and m=4 (**right** column), and average linkage method. In the second row we show the same for distance $D_{V}$. To improve the visibility we write 1 for $x_{n}^{1}$ and so on.

Next, we consider another nonlinear deterministic time series given by the system:

(3) $$\left\{\begin{array}{c} x_{n+1}^{1}=2.25x_{n}^{1}\left(1-x_{n}^{2}\right)\\ x_{n+1}^{2}=x_{n}^{1}\\ x_{n+1}^{3}=x_{n}^{4}\\ x_{n+1}^{4}=x_{n}^{5}\\ x_{n+1}^{5}=4x_{n}^{5}\left(1-x_{n}^{3}\right) \end{array}\right.$$

with initial conditions 0.45 for all the variables. Dendrograms are shown in Figure 7, where as usual we have considered a sample of 10,000 points and the average method. We see that $x_{n}^{1}$ and $x_{n}^{2}$ are connected first and then $x_{n}^{3}$, $x_{n}^{4}$ and $x_{n}^{5}$ are clustered together as one may expect for the system shape. Note that the appropriate clustering is obtained when m=3 and m=4, but not for the case m=2.

### 2.3. Non Linear Dependence: Non Deterministic Systems

**Figure 8:**
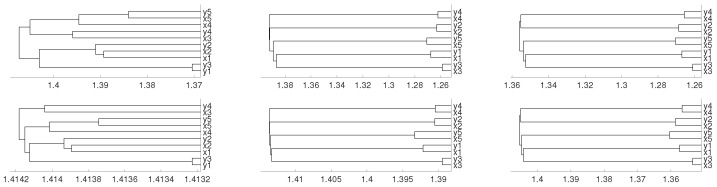
Dendrograms of system (4) for distance *D* (first row) for embedding dimension m=2 (**left** column), m=3 (**central** column) and m=4 (**right** column), and average linkage method. In the second row we show the same for distance $D_{V}$.

First, we consider the time series generated by the difference equations:

(4) $$\left\{\begin{array}{c} x_{n+1}=u_{n}\\ y_{n+1}=0.8{\left(x_{n}\right)}^{2}+v_{n} \end{array}\right.$$

where $u_{n}$ and $v_{n}$ are i.i.d. N(0,1) variables. We consider 0.45 as initial conditions and generate samples of 60,000 points for each variable. Then we divide them into five time series of 12,000 points labeled by $x_{1}$,…, $x_{5}$, $y_{1}$,…, $y_{5}$ in Figure 8, where the results are presented with the average linkage method. We see that embedding dimensions m=3 and 4 give us the expected results.

**Figure 9:**
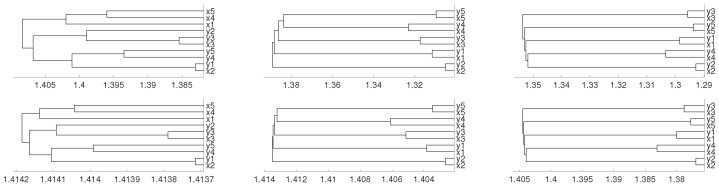
Dendrograms of system (5) for distance *D* (first row) for embedding dimension m=2 (**left** column), m=3 (**central** column) and m=4 (**right** column), and average linkage method. In the second row we show the same for distance $D_{V}$.

Next, we consider a similar example generating 60,000 data points and dividing them into data series of 12,000 points labeled by $x_{1}$,…, $x_{5}$, $y_{1}$,…, $y_{5}$. The points are generated by the system of difference equations:

(5) $$\left\{\begin{array}{c} x_{n+1}=0.6x_{n}+u_{n}\\ y_{n+1}=0.6{\left(u_{n}\right)}^{2}+v_{n} \end{array}\right.$$

where $u_{n}$ and $v_{n}$ are i.i.d. N(0,1) variables. We consider 0.45 as initial conditions and the results are given in Figure 9 for average method. We see how embedding dimensions m=3 and 4 give us the results that one can expect.

**Figure 10:**
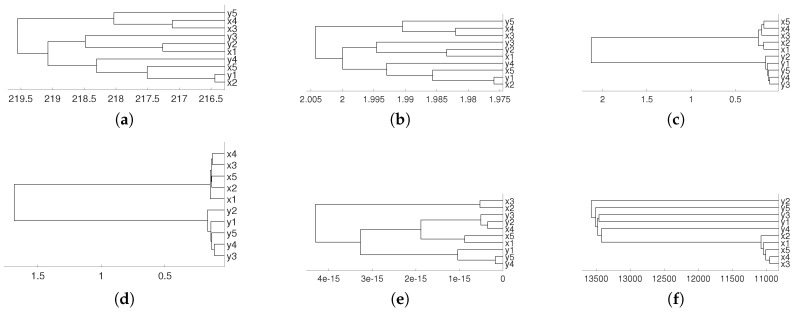
Dendrograms for time series given by the system defined in (5), average linkage and distances: (**a**) Euclidean, (**b**) Pearson’s correlation, (**c**) autocorrelation, (**d**) partial autocorrelation, (**e**) Wavelet Transform and (**f**) Dynamic Time Warping.

Simulated time series given by (5) have also been clustered using traditional distance measures. Figure 10 depicts the results obtained for the Euclidean, Pearson’s correlation, autocorrelation-based, Wavelet Transform and Dynamic Time Warping distances. In the first case, the Euclidean distance does not provide the expected clustering results, because the goal of this distance is to find similar time series in time. In the second case, the correlation-based distance is not able to detect the non-linear relationships among the time series. Regarding the autocorrelation-based distance, which computes the dissimilarity between two time series as the distance between their estimated simple or partial autocorrelation coefficients, two groups are distinguished (one cluster formed by the time series split from $x_{n}$ and the other formed by the time series split from $y_{n}$). Once again, the Wavelet Transform distance does not achieve the correct clustering results. Finally, the Dynamic Time Warping distance is not appropriate for clustering time series by dependency (recall that this distance leads to a shape-based approach in time series clustering, where the time series with similar shape are put together in the same cluster; however, two time series with different shapes can be strongly dependent).

## 3. Real Data Experiments

The above section introduced several simulated time series proving that the distances *D* and $D_{V}$ are useful tools to establish what time series are closer for both linear and nonlinear dependencies. In this section we apply the theoretical framework for real time series.

### 3.1. Latin American Exchange Rate Dependencies

**Figure 11:**
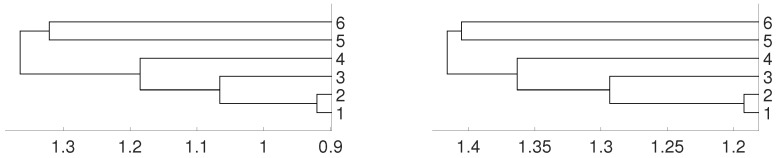
Dendrograms of six Latin American currencies for distance *D* (left) and $D_{V}$ for embedding dimension m=3. The currencies are labeled by 1 for BRL, 2 for MXN, 3 for CLP, 4 for COP, 5 for PEN and 6 for ARS.

We consider the log–returns of daily exchange rates of six Latin American currencies vs. US dollar. Namely, we consider the currency of Argentina (ARS), Brasil (BRL), Chile (CLP), Colombia (COP), Mexico (MXN) and Peru (PEN), from 22 June 2005 to 25 April 2012. The data length is 1754, and it has been already considered in paper [32]. In that paper, authors evaluate the level of contagion among the exchange rates of the previous six Latin American countries, and using copulas they conclude that two blocs were distinguished: the first bloc consists of Brazil, Colombia, Chile and Mexico, whose exchange rates exhibit the largest dependence coefficients, and the second bloc consists of Argentina and Peru, whose exchange rate dependence coefficients with other Latin American countries are low. Figure 11 shows that the same conclusion can be obtained using the method proposed in this paper with embedding dimension m=3 and complete linkage.

### 3.2. Tumor Clustering According to RNA Sequences

The data analyzed below consists of 50 time series of length 20,531 (RNA-sequence). Each series belongs to one of the five types of tumors which can be found and downloaded at the web page https://archive.ics.uci.edu/ml/datasets/gene+expression+cancer+RNA-Seq (see also https://www.synapse.org/#!Synapse:syn2812961). In fact, the original dataset consists of 800 time series, but we have selected the first 10 series of each type of tumor (a total of 50 time series) in order to get a simpler dendrogram. This collection of data is part of the RNA-Seq (HiSeq) PANCAN data set, it is a random extraction of gene expressions of patients having different types of tumors: BRCA (breast carcinoma), KIRC (kidney renal clear-cell carcinoma), COAD (colon adenocarcinoma), LUAD (lung adenocarcinoma) and PRAD (prostate adenocarcinoma).

**Figure 12:**
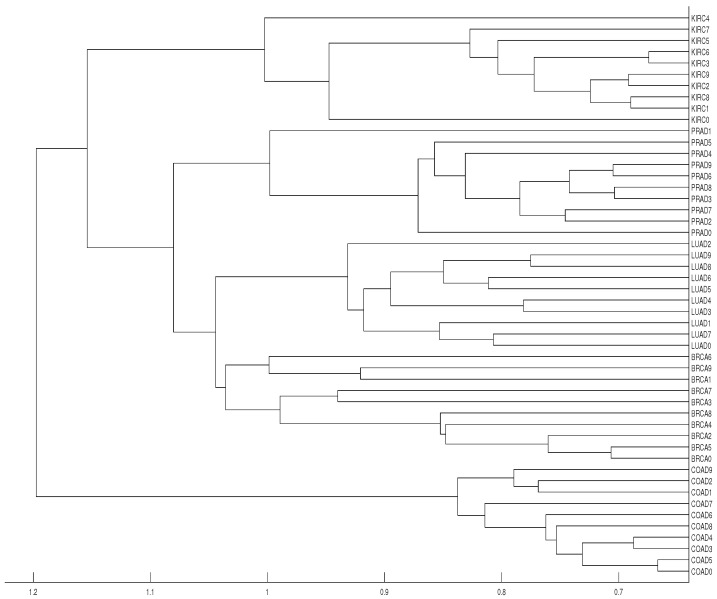
Dendrograms of the 50 time series of RNA-sequence using distance *D*, embedding dimension m=3 and average linkage method.

**Figure 13:**
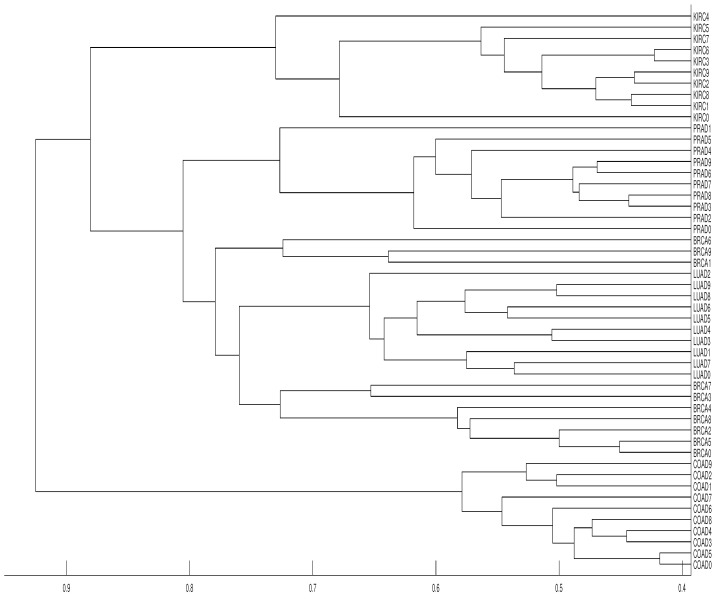
Dendrograms of the 50 time series of RNA-sequence using distance $D_{V}$, embedding dimension m=3 and average linkage method.

Our results show that distance $D_{V}$ clusters properly each data series with its tumor group for embedding dimensions m=3 and average linkage, although the most similar pair of tumors (BRCA and LUAD) cannot be properly grouped. With the same conditions for distance *D*, a completely correct clustering is obtained (see Figure 12 and Figure 13).

### 3.3. Evolution of Spanish IBEX35 Banks

**Figure 14:**
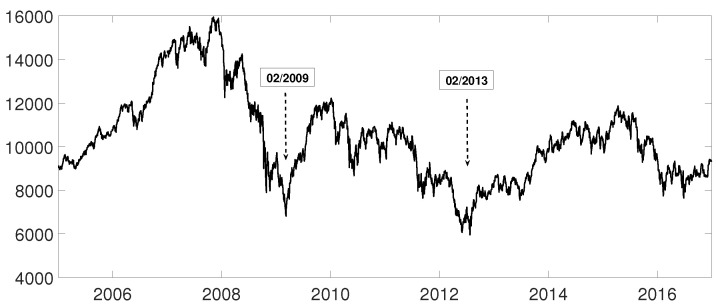
Spanish Ibex35 index evolution from January 2005 to December 2016. The two minimums shown are used to divide the time series.

**Figure 15:**
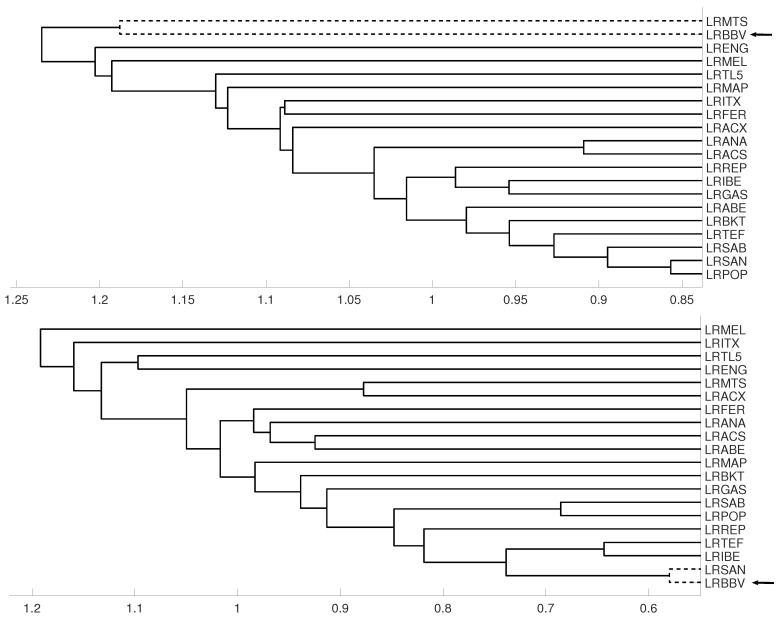

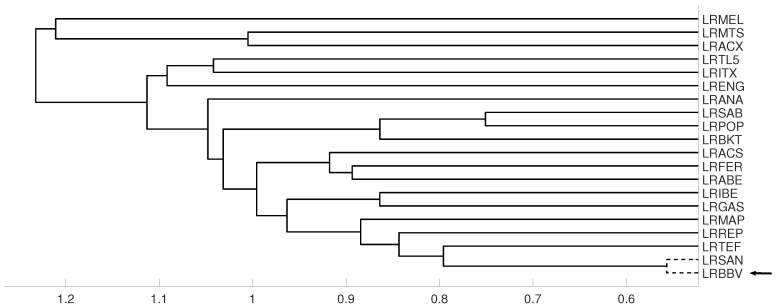
For measure D, embedding m=3 we show the dendrograms in three different periods, from January 2005 to February 2009 (**top**), from: February 2009 to February 2013 (**middle**) and for February 2013 to December 2106 (**down**). Note the evolution of log-returns (LRBBV) with respect to the log-returns (LRSAN).

**Figure 16:**
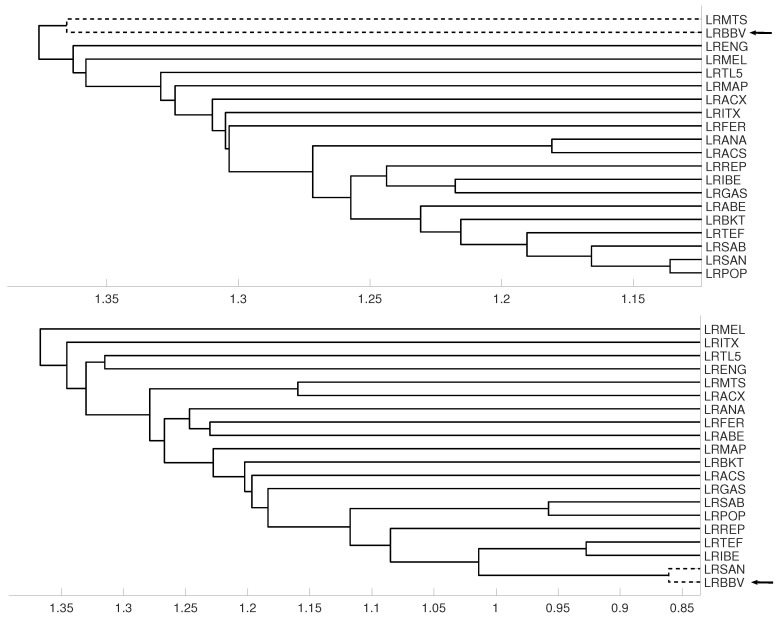

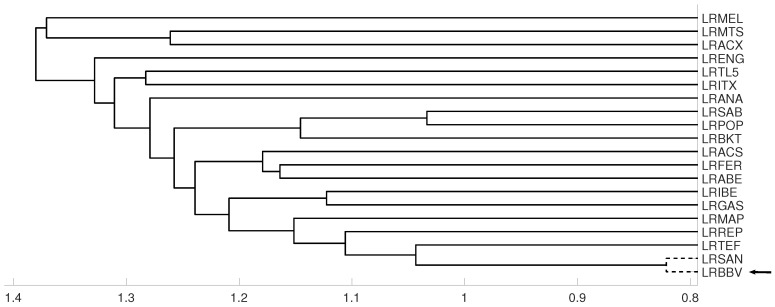
For measure $D_{V}$, embedding m=3 we show the dendrograms in three different periods, from January 2005 to February 2009 (**top**), from: February 2009 to February 2013 (**middle**) and for February 2013 to December 2106 (**down**).

An interesting application consists in analyzing the evolution of log-returns of Spanish banks at IBEX35 Spanish index. In Figure 14 we show the index evolution and the days we use to divide the data. Namely, before the 2008 financial crisis, from 2005 to February of 2009, Banco Popular (POP), Banco Santander (SAN), Banco de Sabadell (SAB) and Bankinter (BKT) were grouped while Banco Bilbao Vizcaya (BBV) was not grouped with them as Figure 15 and Figure 16 and show. After the 2008 financial crisis, from February of 2009 to February of 2013, Banco Santander (SAN) was close to Banco Bilbao Vizcaya (BBV), while Popular (POP), Banco de Sabadell (SAB) and Bankinter (BKT) were in another group as Figure 15 and Figure 16 show. For the last period from February 2013 to December 2016, no changes were found.

## 4. Conclusions

This paper proposes the using of permutations as an efficient tool in time series clustering. Although traditional approaches (shape-based, feature-based and model-based) are useful to cluster time series that are not related among them, we show that permutations play an important role to cluster time series according to their degree of dependency.

Two distances based on permutations have been considered for the simulations, as well as three different embedding dimensions and three linkages methods for the hierarchical procedure. Simulation results demonstrate that:

- The proposed clustering approach is able to detect linear and non-linear dependencies among time series.
- In some cases, a very small embedding dimension like m=2 is not enough to detect dependencies among time series, thus a greater embedding dimension is required.
- The distance measure based on the mutual information has revealed a better performance than the distance measure based on the Crammer’s V statistic.
- There are not significant differences with respect to the selected linkage method.

The necessity of the proposed approach to detect dependencies among time series has been shown using simulated data, by comparing the results obtained with some traditional distances. Furthermore, the performance of this clustering approach has also been validated using real data in the fields of Finance and Health Science.
